# Structure of a C1/C4-oxidizing AA9 lytic polysaccharide monooxygenase from the thermophilic fungus *Malbranchea cinnamomea*

**DOI:** 10.1107/S2059798321006628

**Published:** 2021-07-29

**Authors:** Scott Mazurkewich, Andrea Seveso, Silvia Hüttner, Gisela Brändén, Johan Larsbrink

**Affiliations:** a Wallenberg Wood Science Center, Division of Industrial Biotechnology, Department of Biology and Biological Engineering, Chalmers University of Technology, SE-412 96 Gothenburg, Sweden; b Department of Chemistry and Molecular Biology, University of Gothenburg, SE-405 30 Gothenburg, Sweden

**Keywords:** lytic polysaccharide monooxygenases, LPMOs, auxiliary activity family 9, AA9, *Malbranchea cinnamomea*, enzyme structure

## Abstract

The thermophilic fungus *Malbranchea cinnamomea* contains a host of enzymes that enable its ability as an efficient degrader of plant biomass and that could be mined for industrial applications. This thermophilic fungus has been studied and found to encode eight lytic polysaccharide monooxygenases (LPMOs) from auxiliary activity family 9 (AA9), which collectively possess different substrate specificities for a range of plant cell-wall-related polysaccharides and oligosaccharides. To gain greater insight into the molecular determinants defining the different specificities, structural studies were pursued and the structure of *Mc*AA9F was determined. The enzyme contains the immuno­globulin-like fold typical of previously solved AA9 LPMO structures, but contains prominent differences in the loop regions found on the surface of the substrate-binding site. Most significantly, *Mc*AA9F has a broad substrate specificity, with activity on both crystalline and soluble polysaccharides. Moreover, it contains a small loop in a region where a large loop has been proposed to govern specificity towards oligosaccharides. The presence of the small loop leads to a considerably flatter and more open surface that is likely to enable the broad specificity of the enzyme. The enzyme contains a succinimide residue substitution, arising from intramolecular cyclization of Asp10, at a position where several homologous members contain an equivalent residue but cyclization has not previously been observed. This first structure of an AA9 LPMO from *M. cinnamomea* aids both the understanding of this family of enzymes and the exploration of the repertoire of industrially relevant lignocellulolytic enzymes from this fungus.

## Abstract 2

The crystal structure of the lytic polysaccharide monooxygenase *Mc*AA9F, which is broadly active on both crystalline and soluble glycans and includes an unusual succinimide motif, is reported.

## 1. Introduction   

**Figure 1:**
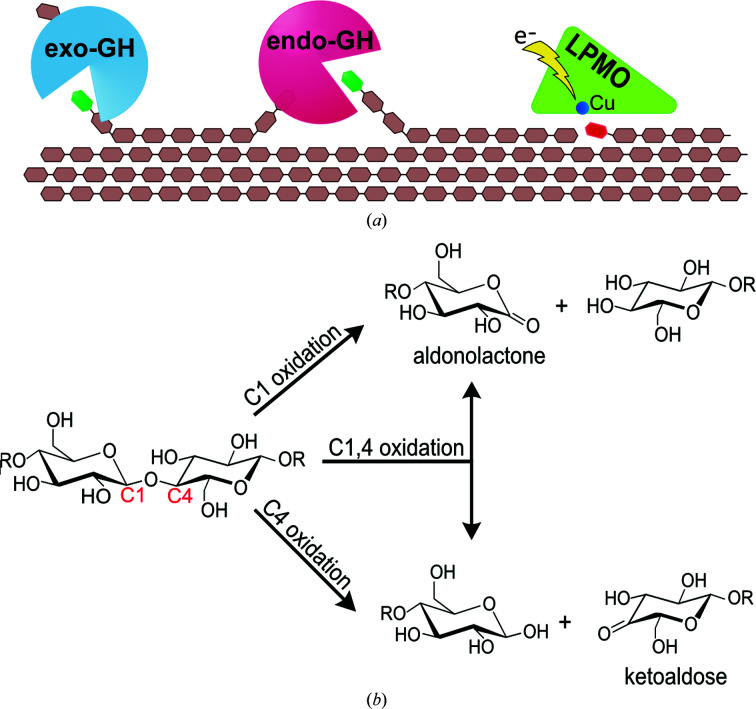
Mode of action of AA9 LPMOs. (*a*) Model for cellulose degradation by LPMOs, which target crystalline regions, and endo- and exo-acting glycoside hydrolases (GHs), which target the ends of glycan chains or amorphous regions, respectively. (*b*) Different types of oxidation of the glycosidic bond performed by AA9 enzymes.

Biorefineries based on the conversion of lignocellulose will play an important role in the move towards a biobased, circular economy, in which renewable plant biomass is converted into valuable products such as fuels, materials and chemical precursors (Ubando *et al.*, 2020 ▸). One significant challenge in the field is the recalcitrance of plant biomass to deconstruction, remodeling or extraction of plant fibers (Sánchez & Cardona, 2008 ▸). Recalcitrance is a multi-factor and multi-scale property emerging from the complex molecular and structural features of the plant cell wall (McCann & Carpita, 2015 ▸), and polysaccharide crystallinity has been identified as an important factor impeding the decoupling of this intricate architecture (Himmel *et al.*, 2007 ▸). In nature, microorganisms harness a vast repertoire of enzymatic activities to access, deconstruct and utilize plant fibers as carbon sources and thus represent a reservoir of enzymes that could be mined for industrial biomass-deconstruction purposes. Fungi in particular are regarded as efficient plant cell-wall metabolizers and employ a wide range of enzymatic activities in the process. Lytic polysaccharide monooxygenases (LPMOs) are a relatively newly discovered class of enzymes and represent one such important activity as they can target highly recalcitrant motifs in the biomass. These include the flat surfaces of crystalline substrates such as cellulose, where an LPMO can generate new polysaccharide ends that aid in accessibility for further depolymerization by glycoside hydrolases (GHs; Fig. 1 ▸ *a*).

Discovered a little over a decade ago, LPMOs are metalloenzymes that utilize a copper ion to catalyze the oxidative cleavage of oligosaccharides and polysaccharides (Vaaje-Kolstad *et al.*, 2010 ▸; Morgenstern *et al.*, 2014 ▸). Interest in LPMOs has grown steadily since their discovery, especially given their importance in increasing biomass valorization yields when supplementing commercial enzyme cocktails (Harris *et al.*, 2014 ▸). LPMOs belong to the auxiliary activities (AAs) class in the Carbohydrate Active enZymes database (CAZy; http://www.cazy.org; Lombard *et al.*, 2014 ▸) and are divided into seven families on the basis of amino-acid sequence similarity [AA family 9 (AA9), AA10, AA11, AA13, AA14, AA15 and AA16]. While their exact catalytic mechanism remains under debate, LPMOs cleave glycosidic bonds by incorporating an O atom in the substrate (Vaaje-Kolstad *et al.*, 2010 ▸; Bissaro *et al.*, 2017 ▸). Proposed models suggest that these enzymes use activated oxygen species to perform a redox reaction with the transfer of two electrons via the Cu atom coordinated by the so-called histidine brace in the active site. Catalysis is thought to be based on the utilization of molecular oxygen, with electrons delivered by an external donor (Vaaje-Kolstad *et al.*, 2010 ▸; Beeson *et al.*, 2012 ▸), although an alternative mechanism has recently been presented which is instead based on the utilization of hydrogen peroxide (Bissaro *et al.*, 2017 ▸; Hangasky *et al.*, 2018 ▸).

Within the AA class, AA9 has received considerable attention as it contains members with diverse substrate specificities ranging from crystalline cellulose to complex glucuronoxylans and also enzymes that are active on oligosaccharides (Lombard *et al.*, 2014 ▸). AA9 enzymes have exclusively been found in fungi and are categorized into subgroups based on the position of oxidation in their reaction products. These subgroups are C1-oxidizing enzymes that generate aldonic acids as products, C4-oxidizing enzymes that generate ketoaldoses as products, and C1,4-oxidizing enzymes, which are more promiscuous and can perform both C1-type and C4-type oxidations, leading to varying mixtures of the two product types (Fig. 1 ▸ *b*). A phylogenetic analysis of AA9 showed that enzymes with different activities fell into distinct clades, and thus a type 1, 2 and 3 nomenclature for C1-, C4- and C1,4-oxidizing AA9 LPMOs, respectively, was proposed (Vu *et al.*, 2014 ▸). However, the preferred site of oxidation may not follow a strict phylogenetic relationship as several exceptions have been found (Vu *et al.*, 2014 ▸; Hüttner *et al.*, 2019 ▸). Filamentous fungi are regarded as major environmental biomass degraders and often encode a surprisingly large number of predicted AA9 enzymes. For example, *Podospora comata* contains at least 33 unique AA9 genes (Lombard *et al.*, 2014 ▸; Boucher *et al.*, 2017 ▸). While significant steps forward in our understanding of AA9 enzymes have been taken in recent years, the reasons behind their abundance in some genomes, and the molecular basis for their specificities and mechanism of action, require additional in-depth studies.

In a recent study, we sequenced the genome of the thermophilic fungus *Malbranchea cinnamomea*, which was isolated from a municipal waste-treatment factory in Vietnam (Hüttner *et al.*, 2017 ▸). This fungus is able to grow at temperatures above 50°C and has been found to be a great source of carbo­hydrate-active enzymes with potential use in industrial biomass-conversion applications (Mahajan *et al.*, 2016 ▸). *M. cinnamomea* encodes eight AA9 enzymes, several of which were upregulated during growth on wheat bran, xylan and cellulose when compared with glucose, thus indicating important roles for these enzymes in biomass turnover (Hüttner *et al.*, 2017 ▸). In a follow-up study, four of the AA9 LPMOs that were possible to heterologously produce in *Pichia pastoris* (*Mc*AA9A, *Mc*AA9B, *Mc*AA9F and *Mc*AA9H) were biochemically characterized. The LPMOs had different substrate specificities and were collectively active on a range of crystalline and soluble plant cell wall-related polysaccharides, as well as oligosaccharides (Hüttner *et al.*, 2019 ▸). *Mc*AA9 enzymes are diverse in primary structure (sequence identities of between 29% and 61%), and while all were predicted to share the expected immunoglobulin G-like β-sandwich fold, considerable differences were predicted among their active-site surfaces. These differences were also reflected in the oxidation patterns, where *Mc*AA9A, *Mc*AA9B and *Mc*AA9F were predominantly C4-oxidizers, but also produced C1- and C1/C4-oxidized cellooligosaccharides from phosphoric acid-swollen cellulose (PASC), while *Mc*AA9H primarily produced C1-oxidized cellooligosaccharides. *Mc*AA9A, *Mc*AA9B and *Mc*AA9F (but not *Mc*AA9H) were shown to act on soluble tamarind xyloglucan (TXG; β-1,4-linked glucose backbone decorated with α-1,6-linked xylose moieties which can be further appended with β-1,2-linked galactose moieties), with concomitant generation of C4-oxidized oligosaccharides, indicating that these enzymes can cleave between any two glucosyl residues in the xyloglucan backbone independently of the side chains present. Furthermore, only *Mc*AA9A and *Mc*AA9F showed clear activity on soluble cellooligosaccharides (cellohexaose), generating C4-oxidation products. The observation that each *Mc*AA9 enzyme appears to have its own activity profile on soluble and crystalline substrates indicates that they each have a different biological role. The differences in activity are likely to stem from differences in the substrate-binding surfaces, and structural studies can thereby facilitate the identification of the molecular motifs responsible for enzyme–substrate interactions.

In this work, we present the structure of *Mc*AA9F and make comparisons with previously characterized AA9 enzymes, highlighting key differences and exploring the possible determinants defining substrate specificity in *Mc*AA9F.

## 2. Methods   

### 2.1. Protein crystallization   

*Mc*AA9F was produced heterologously in *Pichia pastoris* SMD1168H and purified by anion-exchange chromatography as described previously (Hüttner *et al.*, 2019 ▸). The protein was dialyzed into 50 m*M* tris(hydroxymethyl)aminomethane buffer pH 8.0 containing 50 m*M* NaCl. Crystallization conditions were screened with a Mosquito robot (SPT Labtech) using the JCSG+ screening kit (Molecular Dimensions) in MRC sitting-drop plates. Screens were set up with a reservoir volume of 40 µl and protein mixed with reservoir solution in a 1:1 ratio in 0.6 µl drops. After six months, needle-like crystals formed in a condition (C1) which was further optimized to yield clusters of needles with increased dimensions that grew after four months. The final crystallization condition utilized consisted of 100 m*M* phosphate–citrate buffer pH 4.2 with 100 m*M* NaCl, 23.5% PEG 8000. A crystal was separated from the cluster, mounted and flash-cooled in liquid nitrogen in the absence of additional cryoprotectant.

### 2.2. Data collection and structure determination   

A data set was collected to beyond 1.38 Å resolution from a crystal on the BioMAX beamline at MAX IV Laboratory on 11 June 2020. The data set was processed in *XDS* (Kabsch, 2010 ▸) and the structure was determined by molecular replacement with *Phaser* (McCoy *et al.*, 2007 ▸) in *Phenix* (Adams *et al.*, 2010 ▸) using the model of an LPMO from *Aspergillus fumigatus* (*Af*AA9B; PDB entry 6ha5; Lo Leggio *et al.*, 2018 ▸) as the search template. The initial phases were sufficient to enable direct manual rebuilding in *Coot* (Emsley *et al.*, 2010 ▸). *Coot* and *phenix.refine* (Afonine *et al.*, 2012 ▸) were used in iterative cycles of real-space and reciprocal-space refinement. The electron density is well defined, with only the last two residues (Ser221–Gly222) being unable to be resolved in the final model. The data-collection, processing and refinement statistics can be found in Table 1 ▸.

## 3. Results   

### 3.1. *Mc*AA9F structure   

Currently, more than 700 sequences encoding putative AA9 LPMOs can be found in CAZy, although protein structures of only 16 members have been determined (Lombard *et al.*, 2014 ▸). To reveal the key determinants of the different substrate specificities that we observed for the AA9 LPMOs from *M. cinnamomea* (Hüttner *et al.*, 2019 ▸), we pursued structural studies of the enzymes and were able to generate crystals of *Mc*AA9F. The protein crystals grew as needle clusters; a data set was collected to 1.38 Å resolution and the structure was solved by molecular replacement.

**Figure 2:**
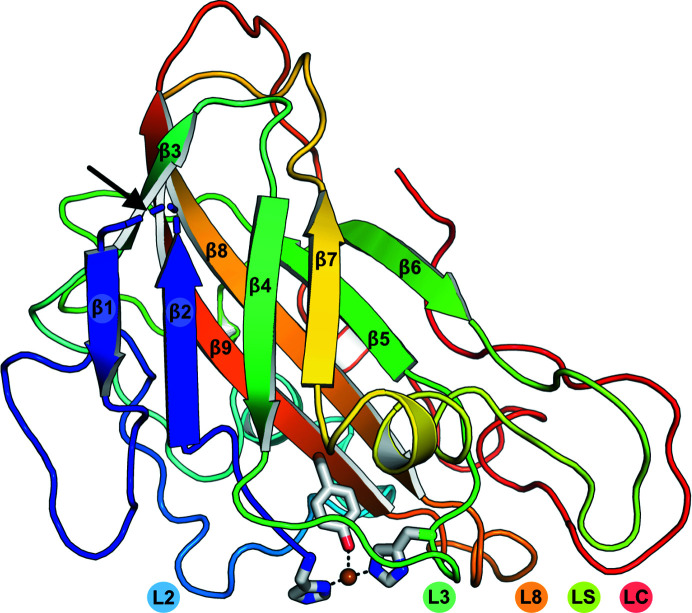
Overall structure of *Mc*AA9F. The structure is colored blue to red from the N-terminus to the C-terminus. The copper ion and the residues coordinating it are shown. The position of the succinimide found in place of Asp10 in the loop between strands β1 and β2 is identified by a black arrow and the loops forming the flat surface comprising the substrate-binding face are labeled (L2, L3, L8, LS and LC).

The overall structure of *Mc*AA9F is comprised of an immunoglobulin-like topology with a central β-sandwich and some small peripheral helical sections, as is typical of previously solved AA9 structures (Fig. 2 ▸). The two β-sheets are composed of four and five β-strands, respectively, which are interconnected by several loops. The loops referred to as L2, L3, LS, L8 and LC (Wu *et al.*, 2013 ▸; Borisova *et al.*, 2015 ▸) participate in forming the flat surface typical of LPMOs acting on crystalline substrates, which also hosts the copper-binding site. Within L2 there are two 3_10_-helices (η-helices) separated by a turn. Another η-helix can be found in the long C-terminal loop LC, while a short α-helix is present in the loop denominated LS. A disulfide bridge between Cys51 and Cys171, which is conserved amongst all structurally determined AA9 LPMO structures, connects loop L2 to strand β9, while another disulfide bridge between Cys92 and Cys96 anchors a short unstructured region located after β6. A fluorescence scan revealed the presence of a copper ion in the crystallized protein, which as expected was found to be co­ordinated by the hydroxyl of Tyr169 and the histidine brace: N^δ^ of His1 and N^ɛ2^ of His81 (Supplementary Fig. S1). Fungal LPMOs are reported to carry an unusual methylation of the N^ɛ2^ atom of the N-terminal histidine residue of the histidine brace, which has been suggested to convey protection against oxidative damage (Petrović *et al.*, 2018 ▸; Quinlan *et al.*, 2011 ▸). This post-translational modification is not seen in *Mc*AA9F, and is also not expected from heterologous protein production in *P. pastoris* (Petrović *et al.*, 2018 ▸). A succinimide is observed in place of Asp10, which is found in a turn connecting β1 and β2 (Supplementary Fig. S2). Succinimides can form as a result of a spontaneous cyclizing dehydration resulting from nucleophilic attack by the main-chain N atom on the γ-carbon of asparagine and aspartate side chains (Haley *et al.*, 1966 ▸; Geiger & Clarke, 1987 ▸). It is extremely rare to find such a motif in protein structures; it has currently been found in only 44 protein entries deposited in the PDB, and to date it has not been encountered in other LPMO structures.

### 3.2. Comparison with other structurally determined AA9 LPMOs   

**Figure 3:**
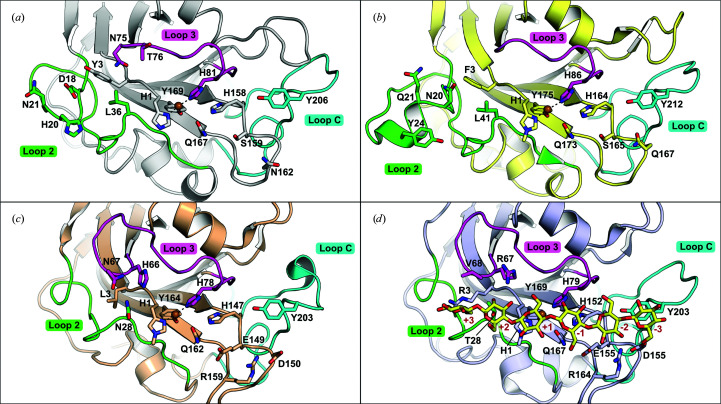
Comparison of the substrate-binding sites of *Mc*AA9A and selected AA9 LPMOs. Key residues lining the flat surface of (*a*) *Mc*AA9F, (*b*) *Ta*AA9A (PDB entry 2yet), (*c*) *Ls*AA9A (PDB entry 5acf) and (*d*) *Cv*AA9A in complex with cellohexaose (PDB entry 6yde) are shown. Loop regions corresponding to L2, L3 and LC for each protein are colored green, magenta and cyan, respectively.

A structural alignment was made using the *DALI* server to aid in the comparison of *Mc*AA9F with other AA9 structures available in the PDB (Holm, 2020 ▸; Supplementary Table S1). The structure-based sequence identities ranged from 31% to 56% and the three enzymes with closest similarity were all predicted C1/C4-oxidizing AA9 LPMOs: *Af*AA9B from *A. fumigatus* (PDB entry 5x6a; 56% indentity; Q. Shen, unpublished work), *Ta*AA9A from *Thermoascus aurantiacus* (PDB entry 2yet; 54% identity; Quinlan *et al.*, 2011 ▸) and *Tr*AA9A from *Trichoderma reesei* (PDB entry 5o2w; 52% identity; Hansson *et al.*, 2017 ▸). The expected conserved overall β-sandwich architecture of AA9 LPMOs was clearly evident, especially the internal β-sheets of the molecule. The active-site geometry is also highly conserved, with a close-to-identical arrangement of the histidine residues forming the brace to coordinate the Cu atom together with the tyrosine residue (Tyr169 in *Mc*AA9F), which is found in an axial position pointing towards the flat surface (Fig. 3 ▸ *a*). Similar to all previously determined AA9 LPMO structures, a glutamine and a histidine residue (Gln167 and His158 in *Mc*AA9F), which have been suggested to be involved in O2 activation, are positioned in proximity to the copper-coordinating residues (O’Dell *et al.*, 2017 ▸; Span *et al.*, 2017 ▸). While containing considerable similarities to other AA9 LPMOs, particularly in the catalytic core, significant differences are observed in *Mc*AA9F, especially in the various loops involved in forming the substrate-binding surface from the N-terminal end of the molecule.

A prominent feature found amongst some C1/C4-oxidizing AA9 enzymes (for example *Ta*AA9A) is the presence of an extended L2 to include a short (six-residue) α-helix that packs parallel to the flat surface and contains a tyrosine residue (Tyr24 in *Ta*AA9A) which has been suggested to be involved in stacking interactions with the substrate (Li *et al.*, 2012 ▸; Fig. 3 ▸ *b*). The extended L2 responsible for this motif was shown to be important for regioselectivity and suggested to govern C4-oxidation in certain AA9 enzymes. A variant of the C1/C4-oxidizing NCU07760 from *Neurospora crassa* (UniProt entry Q7S111) lacking this insert predominantly produced C1-oxidative products from PASC (Vu *et al.*, 2014 ▸). In comparison to *Ta*AA9A, the L2 region in *Mc*AA9F is shorter by five residues and lacks the helical motif. *Mc*AA9F also lacks the conserved tyrosine residue of the motif, but instead possesses a histidine residue (His20), the imidazole side chain of which occupies an equivalent position and is likely to fulfill an equivalent proposed substrate-stacking role. However, the molecular determinants that define C4-oxidative regioselectivity in the AA9 family may in fact not depend upon factors in L2 since other members such as *Cv*AA9A from *Collariella virescens* (PDB entry 5nlt) and *Ls*AA9A from *Lentinus similis* (PDB entry 5acf), two AA9 enzymes with significantly shorter L2 regions, are capable of performing both C1- and C4-oxidation (Simmons *et al.*, 2017 ▸; Frandsen *et al.*, 2016 ▸). A more recent proposal suggests that since similar distances are observed between the C1 and C4 axial protons relative to the active oxygen species in the AA9–substrate complexes, small perturbations in substrate binding may lead to a preferred site of attack, rather than the regioselectivity being more strictly defined by phylogenetic relationships (Simmons *et al.*, 2017 ▸; Frandsen *et al.*, 2016 ▸).

Another characteristic of *Mc*AA9F is that it has a very short L3 loop compared with those found in C4-oxidizing AA9 structures (*Nc*AA9A, PDB entry 5foh; *Nc*AA9C, PDB entry 4d7u; *Nc*AA9D, PDB entry 4eir; Petrović *et al.*, 2019 ▸; Borisova *et al.*, 2015 ▸; Li *et al.*, 2012 ▸) and in the aforementioned *Ls*AA9A and *Cv*AA9A, which are C1/C4-oxidizing AA9 enzymes that are active on oligosaccharides (Fig. 3 ▸ *c*). The presence of an extended L3 loop, which can even harbor a helix formation, has been associated with a capability to act on soluble substrates such as cellooligosaccharides and xyloglucan (Simmons *et al.*, 2017 ▸; Frandsen *et al.*, 2021 ▸). It has also been observed that a truncation of L3 in *Nc*AA9C abolished the activity on xyloglucan (Laurent *et al.*, 2019 ▸). Furthermore, Frandsen and coworkers showed that in *Ls*AA9A the extended L3 loop contributes to the formation of a ridge protruding from the binding surface, which is involved in the interaction with soluble substrates (Frandsen *et al.*, 2016 ▸). In the same study it was also speculated that the presence of this formation caused a lack of enzymatic activity on crystalline substrates. This would be due to the ridge affecting the flatness of the typical LPMO surface, which is suggested to be one of the key factors for the activity on flat, crystalline fibers (Frandsen *et al.*, 2016 ▸). Interestingly, *Mc*AA9F showed activity on both crystalline cellulose and on soluble substrates while containing an extremely short L3 loop aligned to the surface level, indicating that the absence of an extended L3 is not sufficient to define a lack of activity towards soluble substrates and oligosaccharides.

*Mc*AA9F is the first reported AA9 to contain a succinimide modification. The aspartate of this position (Asp10) is conserved in many other AA9 enzymes or is replaced by an asparagine, which is equally prone to cyclization (Clarke, 1987 ▸; Clarke *et al.*, 1992 ▸; Supplementary Table S1). A proposed model for succinimide formation (Bornstein & Balian, 1977 ▸) suggests the absence of steric hindrance close to the main-chain N atom, which performs the attack on the side-chain carbonyl, as an important requirement for cyclization. In *Mc*AA9F, Asp10 is preceded by an alanine and is followed by a glycine, which fulfills these requirements. However, identical or very similar compositions, with valine or isoleucine substituting for the alanine, are found in the majority of AA9 structures, suggesting that this *X*DG (or *X*NG) pattern in the sequence alone might not be sufficient. The occurrence and formation of succinimides can indeed vary depending on the environmental conditions, such as buffer composition (Geiger & Clarke, 1987 ▸; Hooi *et al.*, 2013 ▸), and it is possible that the observed succinimide in the structure of *Mc*AA9F is a result of the crystallization conditions or of the protein-production process.

### 3.3. Comparisons to LPMOs with bound ligands   

In the crystal form of *Mc*AA9F that we obtained, the putative substrate-binding surface is heavily hindered by crystal contacts and thus it would not be possible to pursue ligand complexes using this crystal form (Supplementary Fig. S3). To gain greater insights into the potential key substrate-interacting residues in *Mc*AA9F, we compared the structure with those of other AA9 enzymes in complex with ligands. Both *Ls*AA9A (32% structure-based sequence identity to *Mc*AA9F) and *Cv*AA9A (35% structure-based sequence identity to *Mc*AA9F) are C1/C4-oxidizing LPMOs with an activity profile similar to that of *Mc*AA9F, and structures of both have been solved in complex with cellohexaose (Frandsen *et al.*, 2016 ▸; Tandrup *et al.*, 2020 ▸), towards which *Mc*AA9F also has activity (Hüttner *et al.*, 2019 ▸). Although they share <40% sequence identity, *Cv*AA9A–cellohexaose (PDB entry 6yde) and *Ls*AA9A–cellohexaose (PDB entry 5aci) are closely related in structure to *Mc*AA9F, with root-mean-square deviations of 1.7 and 1.9 Å, respectively, making them good candidates for comparison.

*Ls*AA9A binds the oligosaccharide from subsites −4 to +2, while in *Cv*AA9A the cellohexaose molecule is bound from subsites −3 to +3; however, the orientations and positions of the glucose monomers are conserved. Many of the key residues that make interactions with the substrate from the +1 to −3 sites are either conserved or functionally similar in *Mc*AA9F, including a highly conserved tyrosine residue in LC (Tyr206 in *Mc*AA9F), which has been suggested in multiple studies to be involved in stacking interactions with the flat pyranose ring of the substrates (Li *et al.*, 2012 ▸; Harris *et al.*, 2010 ▸; Asensio *et al.*, 2013 ▸; Nishio, 2011 ▸) and is likely to support substrate binding in a similar fashion in *Mc*AA9F (Fig. 3 ▸ *d*). Greater differences in *Mc*AA9F are present in the +2 sugar-binding site and further towards the substrate reducing-end sites, particularly originating from the differences in L2 and L3 described above. In the extended L3 region of *Ls*AA9A, His66 and Asn67 (PDB entry 5aci) interact with the oligosaccharide by hydrogen bonding to the hydroxyl moieties of C2 and C3 of the sugar at the +2 site. In *Cv*AA9A, leucine and arginine residues (Leu66 and Arg67 in PDB entry 6yde) are found at equivalent positions, with the arginine making a hydrogen bond to the C3 atom of the sugar at the +2 site. While L3 in *Mc*AA9F contains hydrophilic residues capable of interacting with substrates, its considerably smaller size results in the loop being set back >5.5 Å from the +2 sites observed in *Cv*AA9A and *Ls*AA9A. Without considerable rearrangement, it is unlikely that the residues of the short L3 in *Mc*AA9F will be in direct interaction with substrates at the +2 site positions as observed in *Cv*AA9A, *Ls*AA9A and other AA9 LPMOs. Further, in both *Cv*AA9A and *Ls*AA9A a hydrophilic residue in L2 (Asn28 in PDB entry 5aci and Thr28 in PDB entry 6yde) forms an additional hydrogen bond to the C2 hydroxyl of the sugar at the +2 site. The L2 region in *Mc*AA9F is modeled differently and has a leucine residue (Leu36) in the corresponding position with its side chain rotated towards the core of the protein and contributing to creating a flat surface. As previously mentioned, packing of the protein in this lattice leads to considerable contacts across the putative substrate-binding site and may cause some distortions in loop and residue positioning. However, the L2 and L3 regions of *Mc*AA9F are distinct compared with *Cv*AA9A and *Ls*AA9A, which may lead to differences in the positioning of oligosaccharides from the +2 site to the reducing end compared with the other complexes.

## 4. Discussion   

The number of AA9 LPMOs in the genomes of filamentous fungi is often very high, especially in species growing on complex biomass (Lombard *et al.*, 2014 ▸). The multiplicity of AA9 LPMOs in fungal genomes has been highlighted previously (Jagadeeswaran *et al.*, 2016 ▸; Hüttner *et al.*, 2019 ▸; Petrović *et al.*, 2019 ▸), but the reason behind this multiplicity remains unclear. To date, characterization of the full AA9 LPMO repertoire from a single species has not been achieved, which precludes a deeper understanding of their respective biological roles. In many species, however, there are differences in substrate and product specificities among the studied LPMOs, with enzymes acting on both crystalline and insoluble cellulose and soluble heteroglycans, and with C1- and C4- as well as C1/C4-oxidation of the products. *N. crassa*, which is one of the most extensively studied species with respect to its LPMO repertoire, encodes 14 AA9 members in its genome, nine of which have been characterized and shown to be active on cellulose with C1-, C4- and C1/C4-oxidizing activity (Zhou & Zhu, 2020 ▸). Similarly, the thermophilic fungus *M. cinnamomea* encodes eight putative AA9 enzymes, four of which have been biochemically characterized and shown to oxidize a range of both insoluble and soluble substrates with different oxidation patterns (Hüttner *et al.*, 2019 ▸). Curiously, none of the *M. cinnamomea* AA9 enzymes contain appended carbo­hydrate-binding modules, which have been shown to affect regioselectivity in other LPMOs (Chalak *et al.*, 2019 ▸).

The structure of *Mc*AA9F represents the first structurally determined AA9 LPMO from *M. cinnamomea*. The overall fold of the enzyme is similar to those of structurally determined AA9 members, with the typical β-sandwich fold and the expected histidine brace holding the catalytic Cu atom. A peculiar and unexpected feature of the *Mc*AA9F structure is the presence of a succinimide within the protein sequence, which is the first to be reported in a LPMO structure and which originates from the spontaneous cyclization of the aspartate at position 10. No apparent implications for the enzyme activity were observed (Hüttner *et al.*, 2019 ▸), but further structural studies on this and other AA9 LPMOs might help to elucidate the reason behind the formation of such a motif.

One of the most intriguing observations in *Mc*AA9F is the lack of the extended L3 that has been reported to play a major role in interaction with soluble substrates, despite *Mc*AA9F being active on oligosaccharides. A superposition with other structures in complex with cellohexaose showed that certain amino-acid residues that represent contact points with the oligosaccharide substrates do not have an equivalent residue in *Mc*AA9F, suggesting that there might be other regions in *Mc*AA9F that are involved in binding that have not yet been identified and described. Alternatively, cellohexaose could be bound on the *Mc*AA9F surface in a slightly different position, particularly in the +2 site and towards the reducing end, due to the lack of the constraints imposed by the ridge that is observed in members with an extended L3, thus possibly exploiting new points of interaction not described to date. *Mc*AA9F also has activity on the hemicellulosic heteropolysaccharide xyloglucan (Hüttner *et al.*, 2019 ▸). No AA9 LPMO structure in complex with a xyloglucooligosaccharide has yet been obtained and reported, making it difficult to speculate how it would be bound to the surface of the enzyme considering the steric volume represented by the xyloside substituents, although these would be expected to lie in the same flat plane as the glucose-based backbone. The xyloglucan-active *Ls*AA9A and *Cv*AA9A require that xylosyl substitutions occur only on the backbone pyranose monomers at the −3, −2, −1, +2 and +3 subsites, with the +1 subsite occupied by an unsubstituted glucose moiety (Simmons *et al.*, 2017 ▸), while *Mc*AA9A, *Mc*AA9B and *Mc*AA9F can cleave the substrate regardless of the position of the substitutions. These three LPMOs from *M. cinnamomea* all have a predicted significantly shorter L3 loop compared with those exhibited by *Ls*AA9A and *Cv*AA9A (Hüttner *et al.*, 2019 ▸; Supplementary Fig. S4), which suggests that the ridge protruding from the flat surface of the latter enzymes might pose the steric constraints limiting the binding of C6-substituted sugars at the +1 site.

Comparing the sequences of all characterized AA9 enzymes from *M. cinnamomea*, the other type 3 AA9 LPMOs (*Mc*AA9A and *Mc*AA9B) both have a long L2 region similar to that observed in *Ls*AA9A and *Cv*AA9A, with a conserved tyrosine residue likely to aid in binding polysaccharide chains beyond the +2 site (Supplementary Fig. S4). The L2 region in *Mc*AA9F is shorter and is modeled differently but contains a histidine residue that can likely fulfill the functionality of the conserved tyrosine. *Mc*AA9A, *Mc*AA9B and *Mc*AA9F are all active towards PASC, cellooligosaccharides and xyloglucan. Each contains a short L3 region which, as seen in *Mc*AA9F, creates more space around the +2 subsite than is observed in other LPMOs and is likely to contribute to the broad specificity amongst the enzymes. *Mc*AA9H, which lacks activity on both PASC and cellooligosaccharides but has activity towards xylan, lacks the majority of the L2 region, particularly the portion containing the typically conserved L2 tyrosine residue, and has a short L3 region similar to the other characterized AA9 enzymes from *M. cinnamomea*. The determinants resulting in the shift from PASC to xylan activity in *Mc*AA9H remain elusive and further research is needed to explore the molecular features defining this interesting and thus far rare substrate specificity.

## 5. Related literature   

The following reference is cited in the supporting information for this article: Liebschner *et al.* (2017 ▸).

## Supplementary Material

PDB reference: *Mc*AA9F, 7ntl

Supplementary Table and Figures. DOI: 10.1107/S2059798321006628/jc5040sup1.pdf

## Figures and Tables

**Table 1:** Table of crystallographic statistics for *Mc*AA9F

Data collection	
Date	11 June 2020
Source	BioMAX at MAX IV
Wavelength (Å)	0.97625
Space group	*P*12_1_1
*a*, *b*, *c* (Å)	38.96, 43.13, 52.34
α, β, γ (°)	90.00, 101.81, 90.00
No. of measured reflections	220814 (13034)
No. of independent reflections	34403 (2911)
Resolution (Å)	38.13–1.38 (1.43–1.38)
*R*_merge_ (%)	6.38 (87.3)
CC_1/2_ (%)	99.9 (71.5)
Mean *I*/σ(*I*)	13.7 (1.13)
Completeness (%)	92.3 (65.4)
Multiplicity	6.4 (4.5)
Refinement	
*R*_work_/*R*_free_	0.161/0.193
No. of atoms	
Protein	1661
Ligand/ions	22
Water	243
*B* factors (Å^2^)	
Protein	20.3
Ligand/ions	36.9
Water	31.2
R.m.s.d.	
Bond lengths (Å)	0.006
Bond angles (°)	0.91
PDB code	7ntl
