# Supplementary material for: Structure of a C1/C4-oxidizing AA9 lytic polysaccharide monooxygenase from the thermophilic fungus Malbranchea cinnamomea
Source: Acta Crystallogr D Struct Biol. 2021 Jul 29;77(Pt 8):1019–26. doi: 10.1107/S2059798321006628 (PMC8329866; doi:10.1107/S2059798321006628)
Supplement: Supplementary file 1 [file d-77-01019-sup1.pdf]

STRUCTURAL BIOLOGY

Volume 77 (2021)

Supporting information for article:

Structure of a C1/C4-oxidizing AA9 lytic polysaccharide monooxygenase from the thermophilic fungus *Malbranchea cinnamomea*

Scott Mazurkewich, Andrea Seveso, Silvia Hüttner, Gisela Brändén and Johan Larsbrink

Table S1 Comparison of *Mc*AA9F to other structurally determined AA9 enzymes. The list of AA9 enzymes, structurally aligned to *Mc*AA9F using the Dali server, is ordered according to the shown root mean square deviation (RMSD) value and the structure-based sequence identity. The three-residue motif facilitating the formation of the succinimide in *Mc*AA9F is shown along with the corresponding motifs in other AA9 LPMOs.

Organism	Enzyme	PDB accession	RMSD (Å)	Identity (%)	Motif
Malbranchea cinnamonea	*Mc*AA9F	7ntl	/	/	ADG
Thermoascus aurantiacus	*Ta*AA9A	2yet	1.1	54	IDG
Aspergillus fumigatus	*Af*AA9B	6h1z	1.2	55	ADG
Trichoderma reesei	*Hj*AA9A	5o2w	1.2	52	ING
Trichoderma reesei	*Hj*AA9B	2vtc	1.4	46	ING
Neurospora crassa	*Nc*AA9M	4eis	1.5	40	IGG
Heterobasidion irregulare	*Hi*AA9B	5nns	1.6	40	LAG
Collariella virescens	*Cv*AA9A	5nlt	1.6	35	VNG
Neurospora crassa	*Nc*AA9A	5foh	1.6	33	ADG
Neurospora crassa	*Nc*AA9C	4d7u	1.7	35	VNG
Thermothielavioides terrestris	*Tt*AA9E	3eii	1.8	35	NDG
Neurospora crassa	*Nc*AA9D	4eir	1.8	33	VNG
Neurospora crassa	*Nc*AA9F	4qi8	1.8	32	ANS
Lentinus similis	*Ls*AA9B	6rs6	1.8	29	VNG
Lentinus similis	*Ls*AA9A	5acf	1.9	33	VNG
Thermothelomyces thermophilus	*Mt*PMO3	5ufv	2.1	34	IAG
Phanerochaete chrysosporium	*Pc*AA9D	4b5q	2.2	31	PSG

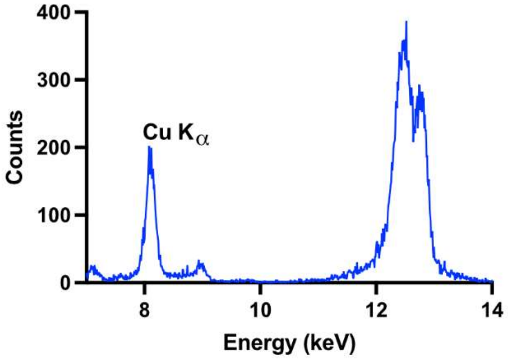

Figure S1 Energy scan of the *Mc*AA9F crystal. A peak corresponding to the copper K_α absorption edge was observed.

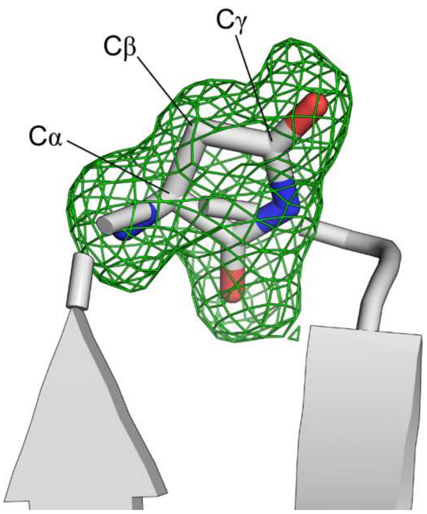

Figure S2 Succinimide observed in place of Asp10 in *Mc*AA9F. Electron density calculated in the form of a polder map (Liebschner *et al.*, 2017) where the succinimide was omitted is displayed around the succinimide and contoured at 3σ.

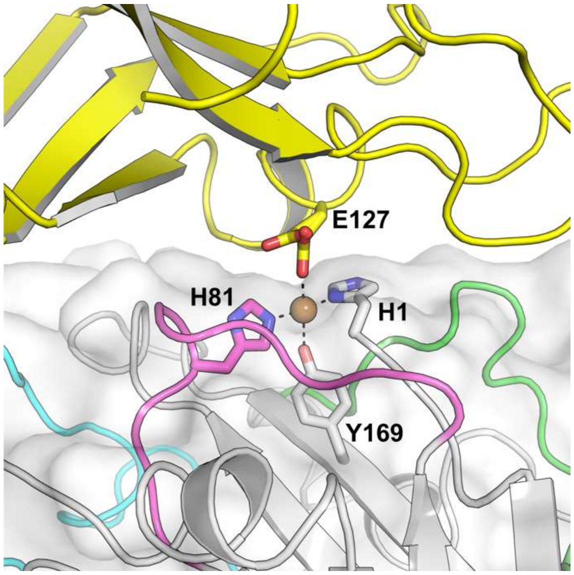

Figure S3 **Crystal packing at the substrate binding face in *Mc*AA9F.** Crystal contacts between a symmetry related molecule (yellow) and the substrate binding face of *Mc*AA9F (coloured as in Figure 3 with the loops L2, L3, and LC coloured green, magenta, and cyan).

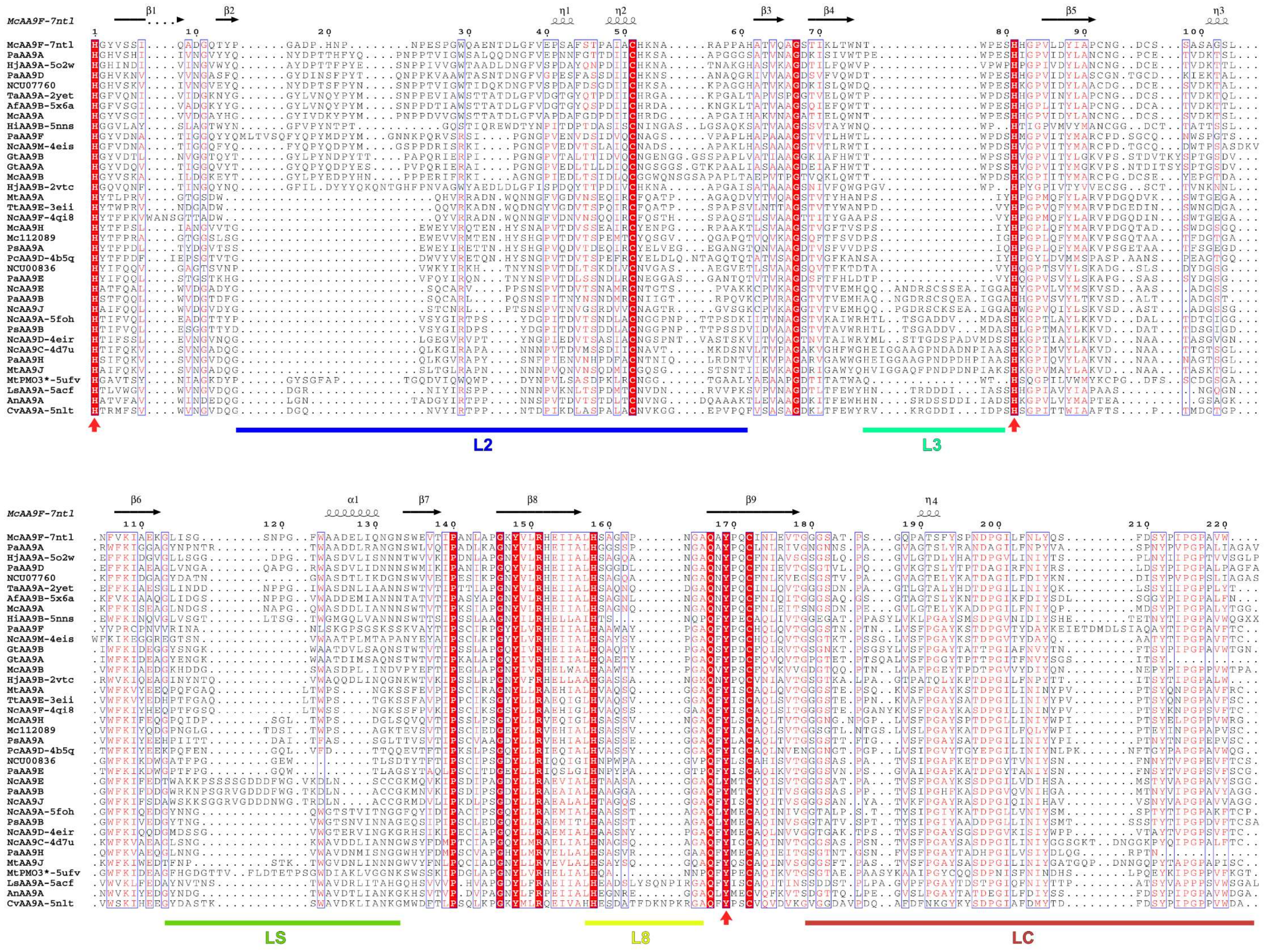
McAA9F-7ntl
β1
β2
η1
η2
β3
β4
β5
η3
McAA9F-7ntl
PaAA9A
HjAA9A-5o2w
PaAA9D
NCU07760
TaAA9A-2yet
AfAA9B-5x6a
McAA9A
HiAA9B-5nns
PaAA9F
NcAA9M-4eis
GtAA9B
GtAA9A
McAA9B
HjAA9B-2vtc
MtAA9A
TtAA9E-3eii
NcAA9F-4qi8
McAA9H
Mc112089
PsAA9A
PcAA9D-4b5q
NCU00836
PaAA9E
NcAA9E
PaAA9B
NcAA9J
NcAA9A-5foh
PsAA9B
NcAA9D-4eir
NcAA9C-4d7u
PaAA9H
MtAA9J
MtPMO3*-5ufv
LsAA9A-5acf
AnAA9A
CvAA9A-5nlt
L2
L3
β6
α1
β7
β8
β9
η4
LS
L8
LC

Figure S4 **Structure-based sequence alignment of AA9 LPMOs.** The sequences of AA9 enzymes biochemically characterized but lacking a structure were aligned with Promals3D to the structure-based alignment of AA9 LPMOs with known structures obtained from Dali. Entries with a structure are noted with a pdb accession. The sequence numbering refers to *Mc*AA9F. Fully conserved residues are shown in white on a red background, blue frames indicate residues with similar physicochemical properties. The typical loop regions of LPMO, named L2, L3, LS, L8 and LC, are marked with blue, cyan, green, yellow and orange respectively. The conserved copper-binding residues are indicated by a red arrow below the alignment. Sequences are listed with the name assigned to the corresponding protein, and the PDB accession code is also written for structurally determined AA9 LPMOs. The sequences shown are from *M. Cinnamomea* (*Mc*), *Podospora anserina* (*Pa*), *T. reesei* (*Hj*), *Neurospora crassa* (*Nc*), *Heterobasidion irregulare* (*Hi*), *L. similis* (*Ls*), *Phanerochaete chrysosporium* (*Pc*), *T. aurantiacus* (*Ta*), *Myceliophthora thermophila* (*Mt*), *Gloeophyllum trabeum* (*Gt*), *Pestalotiopsis sp. NCi6* (*Ps*), *A. fumigatus (Af)*, *Heterobasidion irregulare* (*Hi*), *Thielavia terrestris* (*Tt*), *Aspergillus nidulans* (*An*), and *C. virescens* (*Cv*).

Reference

Liebschner, D., Afonine, P. V., Moriarty, N. W., Poon, B. K., Sobolev, O. V,, Terwilliger, T. C., Adams, P. D. (2017). *Acta Cryst D* **73**, 148-157.
